# Serum Zonulin and Lipopolysaccharide (LPS) Levels in Early Myocardial Infarction: Association with Left Ventricular Ejection Fraction

**DOI:** 10.3390/jcm14176216

**Published:** 2025-09-03

**Authors:** Szymon Olędzki, Arnold Kukowka, Aldona Siennicka, Natalia Jakubiak, Dominika Maciejewska-Markiewicz, Radosław Kiedrowicz, Jarosław Kaźmierczak

**Affiliations:** 1 Department of Cardiology, Pomeranian Medical University, 70-111 Szczecin, Poland; 2 Department of Medical Analytics, Pomeranian Medical University, 70-111 Szczecin, Polandaldona.siennicka@pum.edu.pl (A.S.); 3 Department of Human Nutrition and Metabolomics, Pomeranian Medical University, Broniewskiego 24, 71-460 Szczecin, Poland

**Keywords:** heart-gut axis, lipopolysaccharide, zonulin, heart failure, myocardial infarction

## Abstract

**Background**: Atherosclerosis is the main cause of coronary heart disease, which frequently precedes the onset of heart failure. Lipopolysaccharide (LPS), a pro-inflammatory endotoxin produced by Gram-negative bacteria, exacerbates atherosclerotic processes and negatively impacts myocardial function, particularly in the context of activating low grade inflammation. Zonulin, a key regulator of the blood-gut barrier, modulates tight junction permeability, potentially facilitating the translocation of microbial-derived compounds, including LPS, into the circulation. Given these interactions, we investigated serum levels of lipopolysaccharide and zonulin in patients following myocardial infarction. This study aimed to assess serum levels of zonulin and lipopolysaccharide (LPS) in patients who had experienced a myocardial infarction (MI) and to evaluate the association between these biomarkers and ejection fraction (EF) across different patient groups. **Methods**: 78 patients (22 women and 56 men) diagnosed with MI, who underwent primary PCI (percutaneous coronary intervention), were included in the study. The blood samples were collected between 24 and 48 h after PCI. **Results**: Post-myocardial infarction patients with an ejection fraction ≤ 40% exhibited significantly elevated serum lipopolysaccharide levels compared to those with an ejection fraction > 40%. However, no significant differences in zonulin levels were observed between the study groups. **Conclusions**: Our findings suggest that elevated serum lipopolysaccharide levels may be associated with a reduction in post-infarction ejection fraction. This observation highlights a potential link between endotoxemia and impaired myocardial function following MI, warranting further investigation.

## 1. Introduction

The human gastrointestinal tract hosts approximately 1014 microorganisms, collectively named the gut microbiota [1]. Over the 20th century, extensive research has unveiled the intricate and dynamic relationship between the human host and gut-dwelling microorganisms [2]. This symbiotic relationship, fundamental to human health, has led to the term “superorganism” being used in scientific nomenclature. The gut microbiota exhibits significant variability, both between individuals and within a single individual over a lifetime, influenced by factors such as lifestyle, diet, age, and gender [3]. An individual’s unique microbial composition is referred to as their “enterotype”. Disruptions in the gut microbiota, termed dysbiosis, have been implicated in various diseases, including inflammatory bowel disease cancer, diabetes, and obesity [4,5,6]. Dysbiosis is also increasingly recognized as a contributing factor to cardiovascular diseases, such as hypertension, heart failure, and atherosclerosis [7,8,9]. The gut microbiota plays critical roles in maintaining gut-blood barrier integrity, metabolizing nutrients, synthesizing essential compounds (e.g., vitamin K), fortifying the intestinal epithelium, and modulating immune system function. In return, these microorganisms derive a habitat and essential nutrients from the host [10,11,12].

Emerging research has highlighted the heart-gut axis encompassing molecular and cellular interactions between the gut and the cardiovascular system, particularly focusing on the gut microbiota. Key mechanisms linking the gut and cardiovascular health include gut-blood barrier integrity, chronic inflammation mediated by microbiota-associated molecular patterns (MAMPs), and the synthesis of compounds such as trimethylamine N-oxide (TMAO), which may accelerate atherosclerosis [8,13,14,15,16,17].

### 1.1. Lipopolysaccharide (LPS) and Cardiovascular Health

Among MAMPs, lipopolysaccharide (LPS), a component of the outer membrane of Gram-negative bacteria, is one of the most studied. LPS consists of three primary components: lipid A, a polysaccharide core, and O-antigen side chains. Lipid A is responsible for LPS’s toxic properties, which trigger inflammatory responses [18,19]. Several Gram-negative species are of particular relevance to systemic LPS burden. Members of the Enterobacteriaceae family (e.g., *Escherichia coli*, *Klebsiella pneumoniae*) are major contributors due to their abundance in the gut and potential for translocation under dysbiotic conditions. Opportunistic pathogens such as *Pseudomonas aeruginosa* and *Acinetobacter baumannii* are also recognized producers of highly immunostimulatory LPS, especially in hospitalized or immunocompromised patients. In addition, commensal but pathobiont-associated species such *as Bacteroides fragilis* can contribute to systemic LPS release when intestinal barrier integrity is impaired [20]. The human immune system detects LPS via the Toll-like receptor 4 (TLR4) found on immune cells such as monocytes, neutrophils, macrophages, and dendritic cells [21,22]. Upon recognition, TLR4 activation triggers NF-κB signaling, resulting in inflammation. Serum LPS levels determine the severity of this response: low concentrations may have immunomodulatory effects, while higher levels are associated with inflammation-driven diseases, including atherosclerosis. Extremely high concentrations of LPS can cause fever, tachycardia, or septic shock [22,23,24]. Lipopolysaccharides can also exacerbate atherosclerotic processes through the activation of TLR4 [22,25,26].

### 1.2. Zonulin and Gut-Blood Barrier Integrity

**Figure 1:**
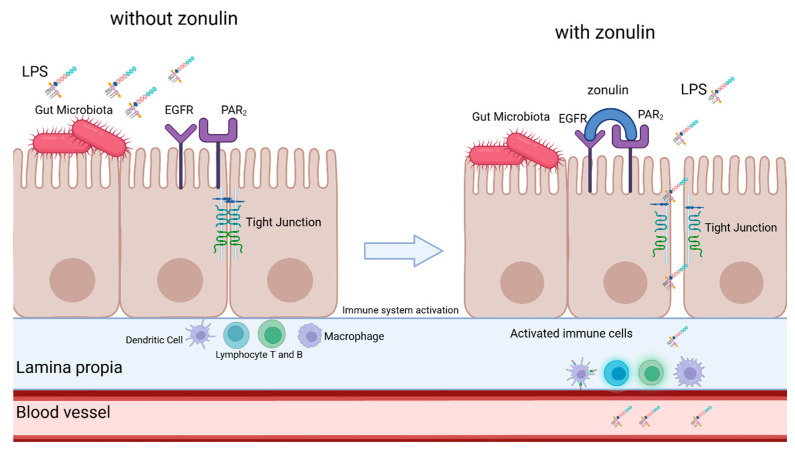
Simplified scheme of zonulin-mediated loosening of tight junctions. Description in the text. LPS—lipopolysaccharide, EGFR—Epidermal growth factor receptor, PAR2—Protease-activated receptor 2. Created in BioRender.

A key element of gut homeostasis is the integrity of the gut-blood barrier, maintained by tight junctions (TJs) between intestinal epithelial cells [27,28]. Damage to TJs increases gut permeability, allowing microbial-derived compounds, including LPS, to enter systemic circulation [29,30,31]. Zonulin, identified in 2001 by Wang et al. [32] is the only known physiological regulator of TJs and modulates gut permeability [29,33,34]. Elevated zonulin levels in intestinal tissue and serum correlate with increased gut permeability and have been associated with diseases such as celiac disease, inflammatory bowel disease, obesity, and neurodegenerative disorders like Parkinson’s disease [35,36,37,38,39,40,41]. Zonulin binds to two receptors—Protease-Activated Receptor 2 (PAR2) and Epidermal Growth Factor Receptor (EGFR)—and upon binding it activates the process of phosporylatiotion of myosin, which is an important supporting protein for TJ’s and the Zonula Occludens-1 protein, which is one component of TJ’s. Another process that is activated by zonulin is the polymerization of actin. As a result of those processes the proteins from which the TJs are composed are displaced, resulting in loosening in the tight junctions (as seen in Figure 1). This creates an opportunity for many antigens such as LPS to bypass the blood-gut barrier and activate the immune system [31,33,34,36].

In chronic heart failure (CHF) the unsealing of the barrier is exacerbated by blood congestion in the intestinal veins—leading to swelling of the intestinal wall, in addition, the intestinal blood supply through the arteries is impaired, which promotes the proliferation of harmful bacteria [8,13,34,42,43]. Given that ischemic heart disease is the most common CHF etiology [44], increased gut permeability has also been detected in this patient population [8,45].

### 1.3. Study Objectives

This study aimed to assess serum levels of LPS and zonulin in patients with acute coronary syndrome, focusing on the relationship between these markers and ejection fraction following a coronary episode.

## 2. Materials and Methods

### 2.1. Patients

Overall, 78 patients (72% men) diagnosed with MI, who underwent primary PCI (percutaneous coronary intervention), were included in the study. The blood samples were collected between 24 and 48 h after PCI. The exclusion criteria are shown in Table 1.

In the studied cohort, all patients (100%) were receiving acetylsalicylic acid. Proton pump inhibitors were used by 27% of patients, while lipid-lowering agents were taken by 43%. 98% of patients were receiving medications related to the treatment of comorbid conditions. Additionally, 38% of patients were on pharmacotherapy aimed at normalizing blood glucose levels. The most common comorbidities were: cardiovascular diseases, dyslipidemia, and diabetes mellitus. The research project was approved by the local ethics committee (KB-006/09/2022, 28 February 2022), and written informed consent was obtained from all participants before the study.

### 2.2. Myocardial Infarction Definition

The diagnosis and therapeutic approach for myocardial infarction followed the recommendations outlined in the 2018 guidelines of the European Society of Cardiology [46]. The study population comprised individuals with both ST-elevation (STEMI) and non-ST-elevation myocardial infarction (NSTEMI).

### 2.3. Serum Collection and Processing

Venous blood samples were collected from all participants using standard phlebotomy procedures. Serum was obtained by collecting blood into clot activator tubes without anticoagulants. After allowing the samples to clot at room temperature, they were centrifuged at 1500–2000× *g* for 10–15 min to separate the serum fraction. All samples were immediately stored at −80 °C until further biochemical analyses were performed.

### 2.4. Biochemical Analysis

Serum concentrations of LPS (EIAab Science Inc., Wuhan, China; 0180) and zonulin (EIAab Science Inc., Wuhan, China; H5560) were determined using commercially available enzyme-linked immunosorbent assay (ELISA) kits. The assays are based on a sandwich ELISA principle: microtiter plates are pre-coated with capture antibodies specific to the analyte of interest. Serum samples and standards are added, allowing antigen binding, followed by incubation with biotin-conjugated detection antibodies. Avidin–horseradish peroxidase (HRP) is subsequently introduced, and the enzymatic reaction is visualized using a chromogenic substrate (TMB). The optical density is measured at 450 nm, and analyte concentrations are calculated relative to a standard calibration curve.

### 2.5. Post-Myocardial Infarction Ejection Fraction

Transthoracic echocardiography (TTE) was performed one day before hospital discharge, typically on the fourth day of hospitalization, and prior to the collection of blood samples for zonulin and LPS measurements. Left ventricular ejection fraction (LVEF) was determined using the biplane Simpson method. Based on LVEF values, patients were classified into two groups: reduced ejection fraction (≤40%) and preserved ejection fraction (>40%).

### 2.6. Statistical Analysis

All statistical analyses were performed using R software (version 4.0.4). The distribution of variables was evaluated with the Shapiro–Wilk test. Since the data did not meet the assumptions of normality, non-parametric methods were employed. Group comparisons were conducted using the Mann–Whitney U test. To assess the relationships between the variables, Spearman’s rank correlation test was applied. A *p*-value < 0.05 was considered statistically significant.

## 3. Results

Baseline characteristics of the study population have been summarized in Table 2. This dataset reflects a typical MI cohort, with mixed systolic function, renal function, and modifiable risk factors, like LDL—cholesterol and BMI.

### 3.1. Zonulin and LPS Status in the Early Phase of MI

Zonulin and LPS status among all patients included in the study irrespective of LVEF are presented in Table 3.

### 3.2. Patient Subgroup Analysis

Among the study participants, 68 individuals exhibited a left ventricular ejection fraction (LVEF) equal to or exceeding 40%. The median LVEF in the preserved-EF group was 51% (interquartile range [IQR] 10%), compared to 34% (IQR 11%) in the low-EF group. No statistically significant differences were observed between the two cohorts regarding baseline parameters such as glomerular filtration rate (GFR), age, or body mass index (BMI). The results are presented in Table 4.

### 3.3. Zonulin and LPS Status According to LVEF

**Figure 2:**
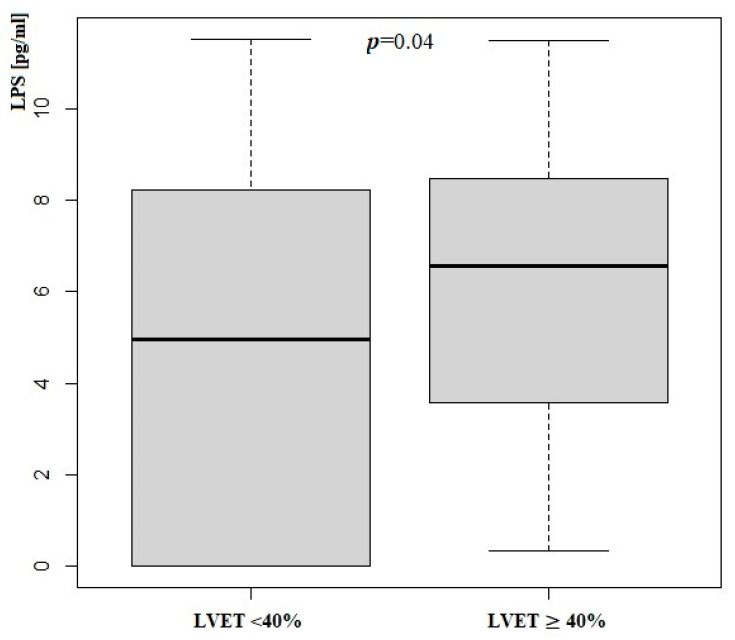
Lipopolysaccharide concentration according to LVEF measurement; LPS—Lipopolysaccharide; LVEF—Left Ventricle Ejection Fraction.

**Figure 3:**
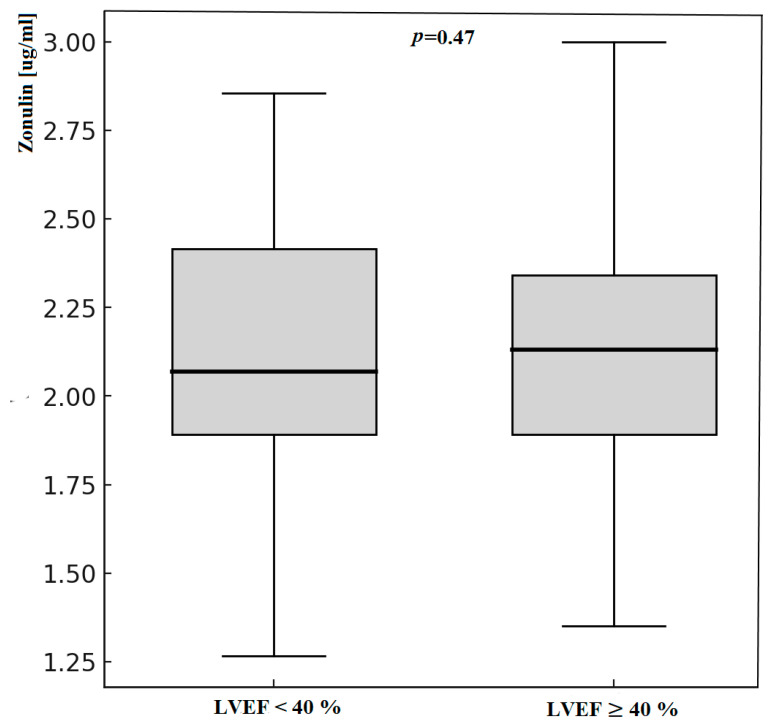
Zonulin concentration according to LVEF measurement; LVEF—Left Ventricle Ejection Fraction.

Patients with LVEF < 40% had significantly higher serum concentrations of LPS. We did not notice any differences in zonulin concentration. Zonulin and lipopolysaccharide status according to LVEF measurement is presented in Table 5 and Figure 2 and Figure 3.

### 3.4. Correlation Between LPS or Zonulin and Anthropometric and Biochemical Parameters

Statistical analysis did not reveal any significant correlations between LPS and zonulin concentrations and the assessed anthropometric or biochemical parameters. All examined correlations were statistically non-significant, with R values below 0.2.

## 4. Discussion

Growing evidence highlights the pivotal role of the gut–heart axis in the pathophysiology of cardiovascular disease. Disruption of intestinal barrier integrity and subsequent translocation of bacterial products such as lipopolysaccharides (LPS) into the systemic circulation may promote systemic inflammation, endothelial dysfunction, and atherogenesis [8]. Zonulin, a modulator of intestinal tight junctions, has emerged as a potential biomarker of impaired intestinal permeability and has been linked to metabolic and cardiovascular disorders. In the context of myocardial infarction (MI), alterations in intestinal permeability and circulating endotoxins may contribute not only to the acute inflammatory response but also to disease progression and adverse cardiac remodeling [9].

In our study, we found that patients with MI had high zonulin and LPS serum levels in general. Thus, we did not compare results with disease-free controls. The results of our study are consistent with previous research findings [47]. Carrera-Bastos et al. analyzed the association between endotoxin (LPS) and zonulin levels and the presence of coronary heart disease. The investigators elucidate that young patients with MI had significantly higher levels of endotoxin and zonulin compared to control groups [47]. High zonulin and LPS levels observed in our study may be the result of systemic inflammatory response to acute coronary syndrome and intestinal motility disorders observed in MI. However, it is not possible to exclude that low-grade immune-mediated inflammation due to dysregulated barrier permeability had an initial impact on the occurrence of an acute coronary event [47]. 

Serum samples were collected only once within the first 48 h after PCI and only. Thus, further studies are needed to analyze dynamic changes in intestinal permeability and the role heart-gut axis in the course of MI. In addition, we found that low EF was associated with increased LPS serum levels. Zonulin concentration, the second marker of inappropriate intestinal permeability, did not differ between patients with low EF and preserved EF. The relationship between low EF and endotoxemia appears to be a predictable result. Patients with decreased EF, especially in the course of acute heart failure, have increased systemic congestion and hypoperfusion, which leads to increased intestinal permeability [48]. On the contrary, Perticone et al. compared 80 HF patients (both preserved and reduced ejection fraction) with 20 healthy controls and found that HF patients had lower endotoxin and zonulin serum levels despite higher values of inflammatory biomarkers and Toll-like receptor expression [49]. Researchers explained the unexpected results by the effect of kidney failure and renal permeability to zonulin [49]. In our study, there were no differences in renal function reflected by the GFR. Thus, our analysis is devoid of distractions such as abnormal kidney function. 

As mentioned earlier—LPS may stimulate the development of atherosclerotic plaques. In atherosclerosis, LPS-mediated TLR4 activation on vascular macrophages induces localized inflammation, promoting plaque formation. Additionally, TLR4 receptors on vascular smooth muscle and endothelial cells exacerbate disease progression by increasing chemokine production and adhesion molecule synthesis, respectively [22,25,26]. LPS shown to disrupt intestinal barrier integrity by modulating tight junction signaling. Upon binding to TLR4, LPS activates downstream adaptor proteins such as MyD88, leading to NF-κB–mediated transcription of proinflammatory cytokines including TNF-α and IL-1β. These mediators induce phosphorylation and redistribution of tight junction proteins such as occludin, claudins, and zonula occludens-1 (ZO-1), resulting in increased paracellular permeability [50]. In addition, LPS–TLR4 signaling promotes activation of MAPK pathways (ERK1/2, JNK, p38), further contributing to cytoskeletal reorganization and tight junction destabilization. Collectively, these mechanisms provide a molecular link between endotoxemia, epithelial barrier dysfunction, and systemic inflammation [51]. Elevated LPS levels are also implicated in left ventricular dysfunction observed during septic shock, underscoring its direct impact on cardiac function, including ejection fraction (EF) [24]. Moreover, LPS potentially triggers reactive oxygen species (ROS) generation, which may have an additional adverse effect on atherosclerotic plaque formation and vascular dysfunction [11]. Not only dysregulated intestinal permeability is linked with adverse cardiovascular effects. Alternation in gut microbiome composition by the imbalance of microbial-dependent metabolites like short-chain fatty acids (SCFAs) and Trimethylamine N-oxide (TAMO) may impact the cardiovascular system [52]. SCFA functions include the maintenance of gut barrier integrity, immune modulation, and antiinflammation response [52]. SCAFAs also play a role in blood pressure regulation, lipid metabolism, and glucose metabolism [52]. TAMO in contrast is a pro-inflammatory agent and is probably involved in atherosclerotic plaque formation [52]. It is worth emphasizing that troponin T (TnT) serum concentration was similar in low-EF and preserved-EF patients. TnT levels were measured upon hospital admission and before PCI, therefore, it seems that there were no significant differences in delay from first medical contact to primary PCI between low-EF and preserved-EF patients. Neither, we did not observe significant differences in other important factors between the two subgroups including age, glycated hemoglobin range, LDL-cholesterol concentration, blood morphology, or BMI. However, this study has some limitations. This study does not include important factors like Toll-like receptor expression or high-sensitivity C-reactive protein. Moreover, a multi-time assessment of LPS and zonulin serum concentration would give us more information about the influence of MI on gut permeability.

## 5. Conclusions

It seems that LPS level is associated with post-MI left ventricle function and the severity of MI. This is evidence of the existence of the heart-gut axis. We hope that this short communication will contribute to a better understanding of this axis function.

## Figures and Tables

**Table 1:** Exclusion criteria from participation in the study, ALT—Alanine Transaminase, AST—Aspartate Aminotransferase, GFR—Glomerular Filtration Rate.

Exclusion Criteria
hematologic diseases
liver diseases—AST or ALT > 150 UI/L
kidney diseases—GFR < 30 mL/min/1.73 m^2^
PCI complication
hypersensitivity reactions to antiplatelet drugs

**Table 2:** Patients characteristics. LVEF; LVEF—Left Ventricular Ejection Fraction; hs-TnT—High-sensitivity troponin T; Hba1c—Glycated hemoglobin A1c; LDL—low-density lipoproteins; WBC—White Blood Cells; PLT-Platelets; Hgb—Hemoglobin; RBC—Red Blood Cells; GFR—Glomerular Filtration Rate; BMI—Body Mass Index.

Parameters	All Patients			
	Median	IQR	Mean	SD
Age [years]	66.3	18.21	65.2	11.7
LVEF [%]	49	13.03	52	10.4
TNT [µg/L]	0.11	0.51	0.34	0.45
Hba1c [%]	5.8	0.6	5.2	1.8
LDL [mg/dL]	124.5	62.7	172	89
WBC [g/L]	9.52	3.95	9.08	4.87
PLT [g/L]	221.5	72.5	294	85
Hgb [mmol/L]	9.15	1.15	9.68	1.54
RBC [t/L]	4.6	0.67	4.8	0.82
GFR [mL/min/1.73 m^2^]	82	32	92	46
BMI [kg/m^2^]	27.9	5.59	28.3	4.4

**Table 3:** Zonulin and LPS status among all patients; LPS—lipopolysaccharide.

Parameters	All Patients	
	Median	IQR
Zonulin [µg/mL]	2.15	0.51
LPS [pg/mL]	6.19	5.07

**Table 4:** Patients analysis according to LVEF; LVEF—Left Ventricular Ejection Fraction; hs—TnT—High-sensitivity troponin T; Hba1c—Glycated hemoglobin A1c; LDL—low-density lipoproteins; WBC—White Blood Cells; PLT—Platelets; Hgb—Hemoglobin; RBC—Red Blood Cells; GFR—Glomerular Filtration Rate; BMI—Body Mass Index.

Parametrs	LVEF ≥ 40%		LVEF < 40%		*p*
	Median	IQR	Median	IQR	
Age [years]	67	17.3	57	13	0.69
LVEF %	51	10	34	11	*p* = 0.001
TNT [µg/L]	0.116	0.52	0.213	0.83	0.42
Hba1c [%]	5.8	0.8	5.9	0.32	0.44
LDL [mg/dL]	138	66.5	123.5	45	0.38
WBC [g/L]	9.9	3.84	10.46	5.69	0.32
PLT [g/L]	221	92	221.5	37.5	0.89
Hgb [mmol/L]	9	1.05	9.3	0.77	0.81
RBC [t/L]	4.62	0.74	4.82	0.41	0.73
GFR [mL/min/1.73 m^2^]	81.5	28	81.5	26	0.84
BMI [kg/m^2^]	28.3	5.52	26.23	4.69	0.08

**Table 5:** Zonulin and LPS serum level according to LVEF; LVEF—left ventricle ejection fraction LPS—lipopolysaccharide.

Parameters	LVEF ≥ 40 %		LVEF < 40 %		*p*
	Median	IQR	Median	IQR	
Zonulin [µg/mL]	2.17	0.53	2.2	0.57	0.47
LPS [pg/mL]	4.97	5.75	6.34	5.22	0.04

## Data Availability

The raw data supporting the conclusions of this article will be made available by the authors on request.
